# Endothelial activation and dysfunction in COVID-19: from basic mechanisms to potential therapeutic approaches

**DOI:** 10.1038/s41392-020-00454-7

**Published:** 2020-12-24

**Authors:** Yuefei Jin, Wangquan Ji, Haiyan Yang, Shuaiyin Chen, Weiguo Zhang, Guangcai Duan

**Affiliations:** 1 Department of Epidemiology, College of Public Health, Zhengzhou University, Zhengzhou, 450001 People’s Republic of China; 2 Department of Immunology, Duke University Medical Center, Durham, NC 27710 USA

**Keywords:** Microbiology, Molecular medicine

## Abstract

On 12 March 2020, the outbreak of coronavirus disease 2019 (COVID-19) was declared a pandemic by the World Health Organization. As of 4 August 2020, more than 18 million confirmed infections had been reported globally. Most patients have mild symptoms, but some patients develop respiratory failure which is the leading cause of death among COVID-19 patients. Endothelial cells with high levels of angiotensin-converting enzyme 2 expression are major participants and regulators of inflammatory reactions and coagulation. Accumulating evidence suggests that endothelial activation and dysfunction participate in COVID-19 pathogenesis by altering the integrity of vessel barrier, promoting pro-coagulative state, inducing endothelial inflammation, and even mediating leukocyte infiltration. This review describes the proposed cellular and molecular mechanisms of endothelial activation and dysfunction during COVID-19 emphasizing the principal mediators and therapeutic implications.

## Introduction

In late 2019, an emerging viral pneumonia caused by a novel severe acute respiratory syndrome coronavirus 2 (SARS-CoV-2), termed coronavirus disease 2019 (COVID-19), was first reported in Wuhan, Hubei Province, China.^1,2^ This disease has infected more than 18 million people and lead to more than 500,000 deaths worldwide.^3^ In general, COVID-19 is considered self-limited, and many of those infected have mild symptoms or appear to be asymptomatic; however, some patients develop respiratory failure which is the leading cause of death among infections with COVID-19.^4–6^ To date, no specific drugs and vaccines are yet available for COVID-19, and its pathogenesis remains largely unclear.

Endothelial cells (ECs) mostly exist in the inner layer of all blood vessels and are normally protected by pericytes.^7^ Pulmonary ECs function as the basic barrier between blood and interstitium, accounting for one-third of cells in the lung.^8,9^ The activation and dysfunction of pulmonary ECs are considered signs of ARDS and the primary pathological causes.^10,11^ Accumulating evidence indicates that SARS-CoV-2 infection exerts adverse effects on the endothelium of capillary, which may contribute to COVID-19 pathogenesis by altering the integrity of vessel barrier, promoting pro-coagulative state, inducing endothelial inflammation, and even mediating leukocyte infiltration.^12,13^ Patients with severe or critical COVID-19 admitted in intensive care units frequently present underlying conditions (old age, diabetes, hypertension, and cardiovascular diseases).^5^ These comorbidities are often accompanied by years of chronic endothelial dysfunction.^13,14^ Endothelial activation and dysfunction are suggested to be related to the coagulation cascade.^15^ Established evidence suggests that activation of the coagulation pathway with the possible development of disseminated intravascular coagulation (DIC) is a feature of severe COVID-19^16,17^ that may further result in thrombus formation.^18^ Therefore, it was suggested to name severe pulmonary COVID-19 as the microvascular COVID-19 lung vessels obstructive thromboinflammatory syndrome or MicroCLOTS.^19^

To date, the ongoing global pandemic of COVID-19 still poses a considerable threat to many people.^20^ An improved mechanistic understanding of endothelial activation and dysfunction is of utmost importance. This review describes the possible cellular and molecular mechanisms of endothelial activation and dysfunction in COVID-19, emphasizing the principal mediators and the therapeutic implications.

## Pathophysiology of ECs

**Fig. 1:**
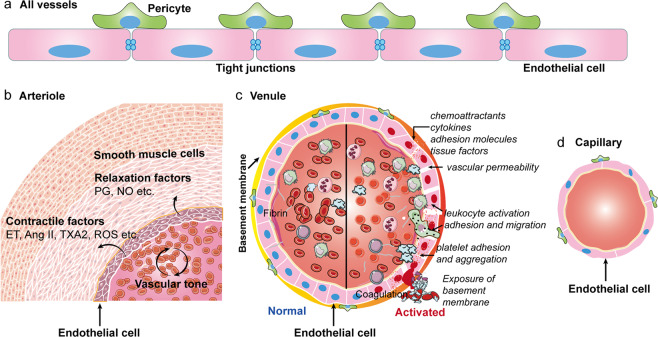
Pathophysiology of endothelial cells. Endothelial cells exist in the inner layer of blood vessels (**a**) such as arteries (**b**), veins (**c**), and capillaries (**d**), and are normally protected by pericytes that support the vessel structure. Tight junctions link neighboring cells and help maintain tissue integrity, act as barriers to permeability. The regulation of vascular tone, and permeability upon activation are illustrated

The primary histological characteristic of resting ECs is their cobblestone shape; however, they constitute more than static mechano-protective plates (Fig. 1).^21^ ECs mostly exist in the inner layer of blood vessels and are normally protected by pericytes that support the vessel structure.^7^ ECs have different functions and structures depending on the tissues and organs. For example, pulmonary vascular ECs are arranged in a dense monolayer, forming a protective barrier that is in direct contact with blood components.^9^ The commonly accepted functions of ECs in the homeostasis of body physiology are controlling vascular permeability and regulating vascular tone (Fig. 1).^21^ ECs can synthesize and release various endothelium-derived relaxation factors, such as nitric oxide (NO) and prostaglandin (PG), and contractile factors, including endothelin (ET), thromboxane A2 (TXA2), reactive oxygen species (ROS), and angiotensin II (Ang II), which play significant roles in the regulation of vascular tone (Fig. 1).^22^ When activated, ECs secrete chemoattractants, cytokines, and adhesion molecules, leading to augmented blood vessel permeability (Fig. 1).^21^ In resting ECs, the synthesis of these molecules can be suppressed by NO.^23^ In addition, ECs are also involved in adhesion and aggregation of platelets, activation, adhesion, and migration of leukocytes, and fibrin balance (Fig. 1).^24^ NO exerts direct effects on leukocytes, preventing their activation into motile forms that are capable of entering tissues.^23^ However, dysfunctional endothelial response to damage or infection cannot produce sufficient amounts of NO.^23^ Therefore, a decline in NO bioavailability always represents endothelial dysfunction.

## Endothelial activation and dysfunction are associated with COVID-19 severity

Epidemiological studies suggest that severe cases or deaths due to COVID-19 frequently present with underlying comorbidities, such as advanced age, hypertension, diabetes, and cardiovascular diseases.^5^ Chronic vascular endothelial injury often co-occurs in patients with such comorbidities.^14^ Most patients with critical COVID-19 die from ARDS, pulmonary edema, cytokine storm, multiple organ failure, and diffuse coagulopathy.^4^ Among the aforementioned causes, ARDS has been considered a result of pulmonary EC damage.^12^ The direct or indirect activation of ECs mediates the extensive production of inflammatory cytokines, adhesion molecules, and chemokines, which may result in cytokine storm, local inflammatory cell infiltration, and vascular leakage.^12,21,23^ The plasma levels of adhesion molecules, such as intercellular adhesion molecule 1 (ICAM-1), fractalkine, vascular cell adhesion molecule-1 (VCAM-1), vascular adhesion protein-1 (VAP-1), and vascular endothelial growth factor (VEGF), had been reported to be elevated among COVID-19 patients, especially in severe patients.^25,26^ Clinical findings also indicate an increased occurrence of Kawasaki disease, a form of vasculitis, in pediatric patients with COVID-19, implying acute vascular inflammation.^27,28^

**Fig. 2:**
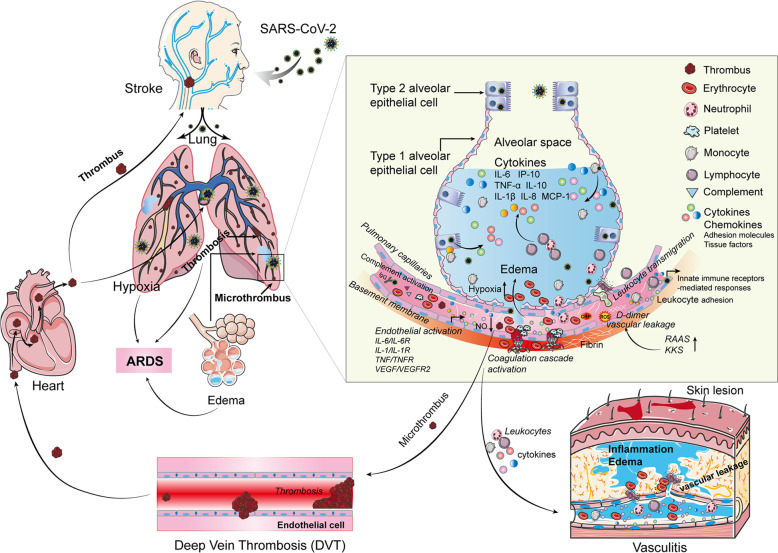
Overview of endothelial activation and dysfunction in the pathogenesis of COVID-19. In the initial stage of severe COVID-19 patients, SARS-CoV-2 infection causes acute lung injury, and then excessive cytokines are released from immune cells, bronchial epithelial cells, and alveolar cells. SARS-CoV-2 infection and various cytokines are predicted to cause endothelial activation and dysfunction by multiple pathways, leading to vascular inflammation and permeability. Then more immune cells enter or migrate into alveoli and enhance lung inflammation. With vascular permeability, erythrocytes enter into alveoli, leading to edema. Moreover, with the release of pro-inflammatory cytokines and inflammatory cells to circulation, vasculitis occurs. The disruption of vascular integrity and EC apoptosis leads to the exposure of the thrombogenic basement membrane and the activation of the clotting cascade. Endothelial cells release relevant cytokines that further augment platelet production. Platelet activation is the primary cause of thrombosis. Inflammation, edema, and microthrombus work together to cause ARDS. The transfer of microthrombi into the blood circulation increases the risk of the formation of deep vein thrombosis, which may further cause pulmonary embolism and stroke

The dysfunction of ECs fails to release sufficient amounts of NO, resulting in vessel constriction.^23^ NO deficiency has been observed among COVID-19 patients,^29^ and it may cause vascular smooth muscle contraction, reducing the ability to neutralize ROS and NO-mediated antiviral capability.^30,31^ ECs function as key regulators of coagulation and as counterbalance for thrombin.^32^ A dysfunctional endothelial response to viral infection can activate the coagulation pathway and lead to anticoagulation imbalance.^7^ Comprehensive coagulation analyzes of patients with COVID-19 indicate elevated levels of D-dimer, increased fibrinogen, enhanced platelet activation, and increased variables of viscoelastic in the plasma of severe cases.^33,34^ Elevated D-dimer levels in critical patients represent poor prognosis.^35^ In addition to venous thromboembolism, the association of microthrombus formation with multiple organ failure and acro-ischemic change has been proposed.^36^ The pathological manifestations leading to severe COVID-19 have been recently considered as vascular leakage, inflammatory reactions, anticoagulation imbalance, and endothelial dysfunction, which may play a central role in the aforementioned procedure.^12^ Therefore, understanding the mechanisms of endothelial activation and dysfunction during the course of COVID-19 infection will help in the early identification of individuals which are at risk of suffering from severe complications, and thus, provide appropriate therapeutic targets. The overview of endothelial activation and dysfunction in COVID-19 pathogenesis is shown in Fig. 2.

## Critical functions of ECs in COVID-19 inflammation

### Innate immune receptor-mediated inflammatory responses in ECs

EC functions can be regarded from two aspects in the immunology of vascular homeostasis and pathology. First, ECs are a constitutive and integral part of the vascular system, and thus intrinsically cause vascular diseases once they are dysfunctional. Second, ECs actively mediate inflammatory or immune responses at infection or injury sites.^21,22^ Innate immunity serves as the first line of defense against microbial pathogens. It depends on a limited number of functional proteins or pattern recognition receptors (PRRs), such as Toll-like receptors (TLRs), nucleotide-binding oligomerization domain (NOD)-like receptors (NLRs), or retinoic acid-inducible gene I (RIG-I)-like receptors (RLRs).^37^ Inflammation is commonly triggered when PRRs detect tissue damage or microbial infection.^37,38^ To date, our knowledge of specific innate immune responses to SARS-CoV-2 in ECs remains unknown. However, the virus–host interactions involving SARS-CoV-2 are likely to recapitulate many of those involving other virus types or microbial infections given the conserved mechanisms of innate immunity. PRR-mediated detection of viral single-stranded RNA (ssRNA) and double-stranded RNA (dsRNA), termed pathogen-associated molecular patterns (PAMPs), and further activation of antiviral innate immune response have been observed during SARS-CoV infection.^39,40^ Existing pharmacological modulators of PRRs mediated inflammatory responses are listed in Table 1.

**Table 1:** Summary of existing pharmacological modulators that act directly or indirectly on innate immune receptors-mediated inflammatory responses in endothelial cells

Potential therapeutic	Formula	Targets	Mechanism of action	Refs
E6446	C_27_H_35_N_3_O_3_	TLR7, TLR9	Inhibit TLR7 and TLR9-mediated deleterious inflammatory responses	^129^
FR 167653	C_24_H_20_FN_5_O_6_S	p38	A potent suppressor of TNF-α and IL-1β production via specific inhibition of p38	^130^
NOD-IN-1	C_18_H_17_NO_4_S	NOD1, NOD2	Mixed inhibitor of NOD1 and NOD2	^131^
Nodinitib-1	C_14_H_13_N_3_O_2_S	NOD1	A NOD1 inhibitor	^132^
GSK717	C_28_H_28_N_4_O_2_	NOD2	Inhibit NOD2-mediated signaling	^133^
Muscone	C_16_H_30_O	NF-κB, NLRP3	Inhibit NF-κB and NLRP3 inflammasome activation and decrease the levels of inflammatory cytokines	^134^
CY-09	C_19_H_12_F_3_NO_3_S_2_	NLRP3	A selective and direct NLRP3 inhibitor	^135^
INF39	C_12_H_13_ClO_2_	NLRP3	An irreversible and noncytotoxic NLRP3 inhibitor	^136^
Ossirene	C_2_H_8_Cl_3_NO_2_Te	IL-1β, IL-10, caspase-1	A potent IL-1β, IL-10 and caspase-1 inhibitor	^132,137^
Mulberroside A	C_26_H_32_O_14_	NLRP3, caspase-1, NF-κB	Inhibit the activation of NLRP3, caspase-1, and NF-κB and the phosphorylation of MAPK exhibiting anti-inflammatory and anti-apoptotic effects	^138^
Desethyl chloroquine	C_16_H_22_ClN_3_	TLRs	An inhibitor of autophagy and TLRs	^139^
Schaftoside	C_26_H_28_O_14_	TLR4, MyD88	Inhibit the expression of TLR4 and MyD88	^140^
Resatorvid	C_15_H_17_ClFNO_4_S	TLR4	Downregulate TLR4-mediated MyD88 or TRIF signaling	^141^

#### TLRs

TLR1~6 and TLR9 have been detected in all types of tissue-specific ECs.^41,42^ Poly (I:C), a synthetic dsRNA analog, can directly activate TLR1/2, TLR3, and TLR4.^41^ Upon activation through TLR, nuclear factor kappa-B (NF-κB) and mitogen-activated protein kinase (MAPK) signaling are initiated *via* myeloid differentiation factor 88 (MyD88) and/or TIR-domain-containing adapter-inducing interferon-β (TRIF).^41^ This process elicits a hyper-inflammatory response by promoting the production of interleukin (IL)-6, IL-8, tumor necrosis factor (TNF)-α, and IL-1β, and adhesion molecules (E-selectin, P-selectin, ICAM, and VCAM). Consequently, vascular permeability is elevated by disrupted junction protein claudin-5 and induction of procoagulant factors, such as tissue factors, plasminogen activator inhibitor type-1 (PAI-1), von Willebrand factor (vWF), and the urokinase plasminogen activator (uPa).^42^ While TLR7/8 is not detected in ECs, human umbilical vein endothelial cells (HUVECs) express TLR3.^43^ ECs also produce type I interferons, such as IFN-α. IFN-α is well known as a significant cytokine during antiviral responses.^43^ Moreover, TLR9 in ECs can be activated when DAMPs, such as DNA and proteins released outside the cell following tissue injury, are recognized.^44^ Once activated, rapid phosphorylation of NF-κB and adhesion molecules E-selectin and ICAM-1-independent MAPK signaling is detected.^44^ In addition, sepsis-related lung inflammation appears to be dependent on the activation of endothelial TLR4-regulating neutrophil sequestration into the lungs.^45^ Collectively, TLRs in ECs are not only important in host defense against infections but they also contribute to microvascular dysfunction and inflammation during systemic infections.

#### NLRs

The NOD proteins NOD1 and NOD2 are two members of NLR family, which function as cytoplasmic PRRs.^41^ Different types of ECs, such as HUVECs, microvascular ECs and human aortic endothelial cells, express NOD1.^41,46^ Upon microbial stimulation, ECs had been shown to produce IL-8 with a NOD1-dependent manner.^46,47^ The NLR proteins containing a PYD (NLRPs) are classified as another NLR subgroup. NLRPs interact with ASC and caspase-1, forming multiprotein complexes called inflammasomes.^48^ Inflammasomes (e.g., NLRP1 and NLRP3) regulate the proteolytic processing of proIL-1β and proIL-18 into mature forms, and an inflammatory cell death termed pyroptosis.^41^ NLRP1/3, ASC, and caspase-1 expression have been confirmed in lung and vascular ECs.^49,50^ The NLRP3 pathway has been recently considered as a novel target for treatment of COVID-19.^51^

#### RLRs

RIG-I and melanoma differentiation-associated gene 5 (MDA5) are two important cytoplasmic sensors for viral RNA, which belong to the RLRs family of PRR.^37,41,42^ RIG-I and MDA5 have been detected ECs (e.g., HUVECs), and RIG-I expression is upregulated upon microbial stimulation.^37,41^ The CARD-containing adapter molecule mitochondrial antiviral-signaling protein (MAVS) is engaged by both RIG-I and MDA5, which in turn stimulate the signaling pathways resulting in IRF3/7-dependent IFN-α/β response and NF-κB-dependent inflammatory genes transcription.^37^

### ECs in adaptive immune responses

Peptide-major histocompatibility complexes (MHCs) and costimulatory molecules are generally required for T cell activation.^52^ All blood vessels are lined by ECs forming a barrier between blood immune cells and parenchymal tissues, and the interaction between T cells and organ resident ECs has mostly remained elusive. Interestingly, a recent study using single-cell transcriptomics found that high levels of genes related to MHC class II-mediated antigen presentation were detected in a subtype of lung capillary ECs,^53^ suggesting a potential role as antigen-presenting cells (APCs) to function in immune surveillance against respiratory pathogens for this EC subtype. However, given the lack of CD80/CD86 costimulatory molecules on the cell surface, ECs are not able to activate naïve T cells. Thus, they probably function as semi-professional APCs.^54^ When reactivated by IFN-γ (mostly derived from Th1 cells) to express MHC molecules, ECs effectively stimulate cytokine production and proliferation of CD4/8 memory T cells.^55^ It was reported that microvascular ECs could stimulate the transendothelial migration of effector memory CD4 T cells.^23^ Additionally, the ECs from chronically inflamed tissues may function in the polarization of inflammatory responses related to adaptive immunity.^23^ Overactivation of T cells, characterized by increased numbers of Th17 cells and hyperactivation of CD8 T cells, had been detected in the peripheral blood of infections with COVID-19.^56^ Taken together, above evidence indicates that the interaction of ECs with T cells may lead to excessive inflammation in severe infections with COVID-19.

### Endothelial adhesion molecule-dependent leukocyte recruitment

Venular ECs form the primary site of leukocyte trafficking from the circulating blood into the tissues. ECs are assumed to participate in leukocytes recruitment from the bloodstream into the sites of infection and inflammation.^23^ During the process of SARS-CoV-2 infection, inflammatory cytokines of IL-1 and TNF-α derived from activated leukocytes, bind to the extracellular domains of IL-1 receptor 1 (IL-1R1) and TNF receptor 1 (TNFR1) on the surface of the endothelial membrane, further initiating various kinase cascades and leading to the activation of NF-κB and activator protein 1 (AP-1).^23^ These transcription factors induce adhesion molecules (ICAM-1, VCAM-1, E-selectin, and P-selectin). VCAM-1 was originally defined as a CD11-/CD18-independent endothelial ligand for mononuclear leukocytes.^23^ It recognizes α4β1 and α4β7 integrins of leukocyte. VCAM-1 is recently emerged as a key inducible EC-expressed adhesion molecule that mediates the recruitment of monocyte to injury and infection sites.^23^ ICAM-1, expressed on the surface of endothelium and in the peripheral vasculature, is upregulated in lesions. Through binding with leukocyte β2 integrins (CD11/CD18), ICAM-1 supports leukocyte arrest and firm adhesion and mediates the transmigration of monocytes and lymphocytes.^23^ Neutrophils and T cells, particularly regulatory T cells (Tregs) in the peripheral blood of humans, possess E‑selectin ligands, and are thus recruited.^23,57^ P-selectin is an adhesion molecule, and mostly expressed on platelets and endothelium, promoting rolling, adhesion, and transmigration of leukocytes by binding to a disulfide-bonded homodimeric mucin-like glycoprotein, P-selectin glycoprotein ligand-1 (PSGL-1) expressed by leukocytes.^58^ P-selectin plays an important role in leukocyte-endothelial interactions, particularly in modulating inflammatory pathways and defense against infections.^58^ As mentioned earlier, the serum levels of adhesion molecules (ICAM-1, VCAM-1, E-selectin, and P-selectin) in severe infections with COVID-19 are significantly increased.^25,59^ Autopsy biopsy of SARS-CoV-2-infected lungs exhibits mononuclear and polymorphonuclear aggregation, accompanied by the apoptotic ECs.^60^ We speculate that these adhesion molecules expressed by ECs mediate inflammatory cell infiltration and EC injury caused by leukocytes contributes to inflammation, particularly in the capillaries during COVID-19 progression. Existing pharmacological modulators that act directly or indirectly on endothelial activation-mediated leukocytes recruitment are listed in Table 2.

**Table 2:** Summary of existing pharmacological modulators that act directly or indirectly on endothelial activation-mediated leukocytes recruitment

Potential therapeutic	Formula	Targets	Mechanism of action	Refs
Phellopterin	C_17_H_16_O_5_	Akt, PKC	Reduce TNF-α-induced VCAM-1 expression and inhibits the adhesion of monocytes to endothelium	^142^
K-7174,K-7174 dihydrochloride	C_33_H_48_N_2_O_6_/C_33_H_50_Cl_2_N_2_O_6_	GATA	Inhibit the expression of VCAM-1 induced by either IL-1β or TNF-α	^143–145^
Natalizumab	Monoclonal antibody	α4β1 integrin	A recombinant, humanized monoclonal antibody, binds to α4β1-integrin and blocks its interaction with VCAM-1	^146^
Gypenoside XLIX	C_52_H_86_O_21_	PPAR-α	A selective peroxisome proliferator-activated receptor (PPAR)-αactivator and inhibits cytokine-induced VCAM-1 overexpression	^147^
ICAM-1-IN-1	C_15_H_11_BrN_2_O_2_S	Integrin	A potent and selective inhibitor of ICAM-1	^148^
A-205804	C_15_H_12_N_2_OS_2_	ICAM-1, E-selectin	A potent and selective lead inhibitor of ICAM-1 and E-selectin	^149^
Lifitegrast (SAR 118)	C_29_H_24_Cl_2_N_2_O_7_S	LFA-1	Inhibit T cell attachment to ICAM-1	^150,151^
RWJ 50271	C_18_H_17_F_3_N_4_O_2_S	LFA-1/ICAM-1	A selective inhibitor of LFA-1/ICAM-1	^152^
BMS-688521	C_26_H_19_Cl_2_N_5_O_4_	LFA-1/ICAM-1	Inhibitor of the LFA-1/ICAM interaction	^153^
Bimosiamose (TBC-1269)	C_46_H_54_O_16_	E-selectin/ P-selectin	A nonoligosaccharide pan-selectin antagonist	^154^
Andrographolide	C_20_H_30_O_5_	NF-κB	Suppress the activation of NF-κB in stimulated endothelial cells	^155^
Sialyl-Lewis X	C_31_H_52_N_2_O_23_	E-selectin	A high-affinity ligand for selectins and inhibits E-selectin-mediated neutrophil recruitment	^156^
PSI-697	C_21_H_18_ClNO_3_	P-selectin	Inhibit the binding of a soluble human P-selectin to PSGL-1	^157^
Parmodulin 2	C_17_H_17_BrN_2_O_2_	PAR1	An allosteric inhibitor of protease-activated receptor 1 (PAR1)	^158,159^

## Endothelial activation and dysfunction are associated with thrombosis formation during COVID-19

Increasing evidence worldwide suggests that patients with severe COVID-19 frequently develop pulmonary embolism (PE), deep vein thrombosis (DVT), stroke, and even thrombosis in the extracorporeal circuits and arterial thrombosis (Fig. 2).^12,61–63^ EC swelling with foamy degeneration and a few areas of segmental fibrin thrombus in glomerular capillary loops were found in patients who have died from COVID-19 likely due to excessive endothelial activation and dysfunction.^60^ The association of microthrombus formation with organ dysfunction and ARDS has been proposed recently.^12,34^ Coagulation pathway activation with the potential development of DIC is a general characteristic of severe infections with COVID-19, and one of the most common findings is the increase of fibrin degradation fragments (D-dimer).^12,33,64^ Coagulation is a highly well-organized procedure that involves the interaction of ECs, platelets, and coagulation factors.^32^ Upon endothelial activation and dysfunction, disruption of vascular integrity and EC apoptosis results in exposure of the thrombogenic basement membrane and activation of the clotting cascade.^7^ In addition, ECs activated by IL-1β and TNF-α can trigger coagulation by displaying vWF, P-selectin, and fibrinogen, onto which platelets bind.^23^ In turn, ECs release relevant cytokines which augment platelet production. Platelet activation is the primary cause of thrombosis.^15^ Platelets also produce VEGF, which promotes ECs to express the tissue factor, i.e., the main activator of the coagulation cascade.^15^ In response, the fibrinolytic system is activated and releases D-dimers into the circulation.^36^ ARDS develops due to the DIC and clogging of capillaries by inflammatory leukocytes and possible thrombosis in larger blood vessels. To date, at least three strategies, namely, heparin for VTE prevention, anticoagulant, and anti-platelet therapies, have been suggested to treat coagulation abnormalities and thrombosis related to endothelial activation and dysfunction (Table 3).^36^

**Table 3:** Potential therapeutic tools for anticoagulant and antithrombotic treatment in COVID-19

Potential therapeutic	Formula	Targets	Mechanism of action	Refs
Heparin	No application	Endogenous metabolite	Exhibits anti-inflammatory effects and protects the endothelial cells by reducing the toxicity of histones, and decreases lung edema and vascular leakage.	^160–162^
Activated protein C	C_91_H_130_N_22_O_23_	Coagulation cascade	A peptide potently inhibits activated protein C anticoagulant activity	^163,164^
Sofigatran	C_24_H_44_N_4_O_4_S	Active factor IIa	An orally active factor IIa (thrombin) inhibitor	^165^
Methylprednisolone	C_22_H_30_O_5_	Glucocorticoid Receptor	A synthetic corticosteroid with anti-inflammatory and immunomodulating properties	^166^
Clopidogrel	C_16_H_16_ClNO_2_S	P2Y12 receptor	A potent antiplatelet agent that inhibits blood clots	^167^
Ticagrelor	C_23_H_28_F_2_N_6_O_4_S	P2Y12 receptor	Inhibits platelet aggregation	^168^
Prasugrel	C_20_H_20_FNO_3_S	P2Y12 receptor	Inhibits platelet aggregation	^169^
Elinogrel	C_20_H_15_ClFN_5_O_5_S_2_	P2Y12 receptor	Inhibits platelet aggregation	^170^

## Proposed mechanisms for endothelial activation and dysfunction in COVID-19

### SARS-CoV-2 infection directly induces EC apoptosis

**Fig. 3:**
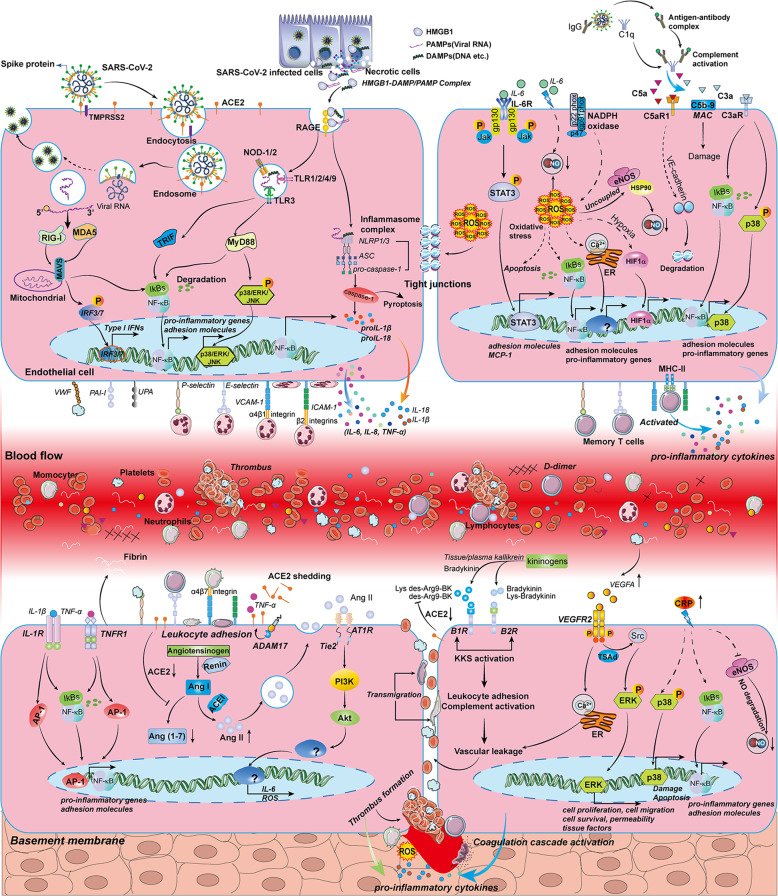
Proposed mechanisms of endothelial activation and dysfunction during COVID-19 This picture highlights possible mechanisms of endothelial activation and dysfunction during SARS-CoV-2 infection, including loss of vascular integrity, vascular permeability, activation of the coagulation pathway, inflammation, and thrombus formation

Established evidence suggests that SARS-CoV-2 hijacks the cell membrane receptor ACE2 to invade host cells with involvement of transmembrane protease serine 2 (TMPRSS2). Human ECs express ACE2 and TMPRSS2 and are considered SARS-CoV-2 target cells.^1,65^ SARS-CoV-2 replication is detected in ECs from various organs of patients with COVID-19 or engineered human blood vessel organoids.^60,66–68^ Autopsy pathology shows the presence of the virus and rupture of the cell membrane of pulmonary ECs.^63^ Moreover, SARS-CoV-2 proliferation in ECs directly induces damage and apoptosis (Fig. 3).^60^ We reviewed several antiviral drugs in our previous publication.^4^ At present, several monoclonal neutralizing antibodies against SARS-CoV-2 have been developed to treat COVID-19 patients.^69,70^

### Loss of ACE2 leads to the imbalance of the renin–angiotensin–aldosterone system (RAAS) and the kallikrein–kinin system (KKS)

ACE2 not only is the receptor for viral entry but also an important component of RAAS.^71^ ACE2 plays a significant role in self-repair of ECs^71^ and the development of acute lung failure caused by SARS-CoV, and other viruses (e.g. avian influenza A strains) by modulating RAAS.^72^ The amount of ACE2 in ECs is decreased as a result of competitive binding and shedding induced by TNF-α and metalloprotease 17 (ADAM17).^73,74^ Consequently, ACE2 fails to catalyze the conversion of Ang II to Ang (1–7), leading to accumulation of Ang II and pathological damage.^75^ Clinical reports have suggested that the level of Ang II in COVID-19 patients is considerably higher than that in healthy individuals.^76^ Ang II is an autocrine vasoconstrictor of ECs.^77^ Excessive Ang II activates the PI3K-Akt signaling pathway through the AT1 receptor to regulate endothelial activation and production of IL-6 and ROS (Fig. 3).^78–81^ In addition, high concentrations of Ang II cause EC death and vascular degeneration by destroying the connection between ECs and pericytes.^82,83^ KKS has been suggested to regulate many physiological processes, such as inflammation, coagulation, vasodilation, and blood pressure. ECs are assumed to be a target of KKS.^84,85^ Under inflammation or infection, the expression of the bradykinin receptors (B1R and B2R) of KKS is upregulated in ECs. However, a decrease in the quantity and activity of ACE2 fails to inactivate the ligands of B1R, Lys des-Arg9-BK, and des-Arg9-BK in the lungs, and in turn, activates KKS (Fig. 3).^86,87^ Activated KKS will cause endothelial dysfunction and further result in leukocyte adhesion, complementing activation.^88,89^ We have summarized potential therapeutic tools for suppressing RAAS and KKS activation (Table 4), except for RAAS inhibitors, including ACE inhibitors and angiotensin II receptor blockers (ARBs), which have been indicated no benefit for COVID-19 protection.^65^

**Table 4:** Summary of existing pharmacological modulators that act directly or indirectly on RAAS and KKS activation during COVID-19

Potential therapeutic	Formula	Targets	Mechanism of action	Refs
Resorcinolnaphthalein	C_24_H_14_O_5_	ACE2	A specific ACE2 enhancer	^171^
SL910102	C_30_H_30_N_6_O	AT1 receptor	A unlabeled nonpeptide AT1 receptor antagonist	^172^
BMS-248360	C_36_H_45_N_5_O_5_S	AT1 receptor	An antagonist of AT1 receptor	^173^
Losartan potassium	C_22_H_22_ClKN_6_O	Ang II	An AT1 receptor antagonist	^174^
Telmisartan	C_33_H_30_N_4_O_2_	AT1 receptor	A long lasting antagonist of AT1 receptor	^175^
Methylprednisolone	C_22_H_30_O_5_	Glucocorticoid receptor	Activate ACE2 and reduces IL-6	^166^
TAPI-1	C_26_H_37_N_5_O_5_	ADAM17	Block the shedding of several cell surface proteins	^176^
Noscapine hydrochloride	C_22_H_24_ClNO_7_	Bradykinin	a non-competitive Bradykinin inhibitor	^177^
SSR240612	C_42_H_53_ClN_4_O_7_S	B1R	A specific non-peptide bradykinin B1R antagonist	^178^
Icatibant acetate	C_61_H_93_N_19_O_15_S	B2R	A specific peptide antagonist of B2R	^179^
Fasitibant chloride	C_36_H_49_Cl_3_N_6_O_6_S	B2R	A selective nonpeptide bradykinin B2R antagonist	^180^

### HMGB1-RAGE/TLR4 signaling-mediated endothelial activation and dysfunction

The high mobility group box 1 (HMGB1), a ubiquitous nuclear protein has been demonstrated to cause endothelial activation and dysfunction.^90,91^ During the process of hypoxic cellular stress or necrosis, HMGB1 is released into the extracellular environment and becomes a lethal pro-inflammatory mediator due to engagement of the receptor for advanced glycation products (RAGE) and TLR4.^92,93^ Upon RAGE or TLR4 activation, adhesion molecules (e.g., ICAM-1, P-selectin, and VCAM-1) and pro-inflammatory cytokines (TNF-α, IL-1, and IL-6) are induced through activating Src kinase, MAPK, and NF-κB.^94–96^ HMGB1 can increase the permeability of endothelial cell monolayer via the RAGE-Src pathway (Fig. 3).^95^ HMGB1 has been considered a potential marker of acute lung injury complicated by ARDS, notably viral pneumonia, such as SARS.^97–99^ Therefore, an increase in HMGB1 is predicted during the progress of COVID-19. At present, HMGB1 has been declared as a therapeutic target for treatment of COVID-19.^100^ The agents that can inhibit HMGB1-RAGE/TLR4 signaling are listed in Table 5.

**Table 5:** Potential therapeutic tools for inhibiting HMGB1-RAGE/TLR4 signaling during COVID-19

Potential therapeutic	Formula	Targets	Mechanism of action	Refs
Glycyrrhizic acid	C_42_H_62_O_16_	HMGB1	A direct HMGB1 antagonist	^181^
Ammonium glycyrrhizinate	C_42_H_65_NO_16_	HMGB1	A direct HMGB1 antagonist	^182^
FPS-ZM1	C_20_H_22_ClNO	RAGE	A high-affinity RAGE inhibitor	^183^
Azeliragon	C_32_H_38_ClN_3_O_2_	RAGE	An inhibitor of RAGE	^184^

### Oxidative stress- and ROS formation-mediated endothelial dysfunction

Oxidative stress is defined as a state of the excessive generation of oxidant compounds and/or the reduction in savaging antioxidants.^101^ Its sequelae include elevated levels of oxidized biomolecules and related tissue damage. Increasing evidence indicates that oxidative stress plays an important role in promoting endothelial dysfunction.^101^ A number of mechanisms may participate in oxidative stress-mediated endothelial dysfunction, but the predominant mechanism is likely to associate with reduced NO bioavailability.^101^ Decreased endothelial nitric oxide synthase (eNOS) expression, lack of substrates for eNOS, eNOS inactivation, and accelerated NO degradation have been considered to cause a decline in NO bioavailability.^101^ As mentioned earlier, the serum level of NO among patients with COVID-19 is decreased, implying oxidative stress.^29^ In general, another main source of oxidative stress is ROS derived from mitochondria.^101^ Physiological ROS generation is required for maintaining the regular vascular homeostasis.^101^ In the vasculature, some enzyme systems, such as NADPH oxidase (NOX) and differentially localized and expressed eNOS, participate in ROS formation. Among these systems, NOX apparently plays a crucial role in orchestrating the activation and dysfunction of other enzymes. This process is considered the major source of ROS in the vascular endothelium.^102^ Acute inflammation by multiple mechanisms contributes to COVID-19 pathogenesis.^40^ Under acute inflammation status, however, excess ROS production can cause oxidation of macromolecules, promoting cell apoptosis mediated by cytochrome-c release.^101^ ROS is also capable of activating calcium signaling and NF-κB signaling to induce adhesion molecules and pro-inflammatory cytokines, which can increase vascular permeability and promote leukocyte adhesion (Fig. 3).^23,103,104^ A recent study suggests that oxidative stress caused by NOX2 activation contributes to COVID-19 pathogenesis and is associated with thrombotic events in COVID-19 patients.^105^ Therefore, a beneficial effect of antioxidant drugs (Table 6) on endothelial function should be considered for the treatment of COVID-19 in the future.

**Table 6:** Potential therapeutic tools for antioxidant treatment during COVID-19

Potential therapeutic	Formula	Targets	Mechanism of action	Refs
Human recombinant IL-37		AMP-activated kinase	Increase NO bioavailability and reduces ROS formation	^185^
DAQ B1		Akt	An activator of Akt, and reduces oxidative stress	^186^
BMOV		PTPase	An inhibitor of PTPase that activates eNOS and reduces oxidative stress	^187^
N-Acetyl-L-cysteine	C_5_H_9_NO_3_S	Endogenous metabolite	A ROS inhibitor	^188^
VAS2870	C_18_H_12_N_6_OS	NOX	A pan NOX inhibitor	^189^
APX-115	C_17_H_18_ClN_3_O	NOX	An active pan NOX inhibitor	^190^
Setanaxib	C_21_H_19_ClN_4_O_2_	NOX1/4	A selective NOX1/4 inhibitor	^191^
gp91ds-tat	C_98_H_190_N_50_O_22_S	NOX	Reduce ROS formation and platelet activation	^192^
GLX351322	C_21_H_25_N_3_O_5_S	NOX4	An inhibitor of NOX4	^193^
GSK2795039	C_23_H_26_N_6_O_2_S	NOX2	A NOX2 inhibitor	^194^

### IL-6-/IL-6R-mediated endothelial activation and dysfunction

Clinical reports have suggested that an increasing level of circulating IL-6 is related to the pathogenesis of COVID-19.^6,106^ IL-6 is produced by multiple cell types that include monocytes/macrophages, adipocytes, and ECs and is elevated in circulation during inflammatory conditions.^107^ Through the engagement of IL-6 receptor (IL-6R), IL-6 initiates the JAK-STAT pathway^80^ and in turn upregulates adhesion molecules, (VCAM-1, ICAM-1, E-selectin), and MCP-1, enhancing leukocyte adherence and extravasation into the vascular wall.^80^ In addition to classical IL-6R signaling, IL-6 is known to reduce NO bioavailability and increase oxidative stress, leading to endothelial permeability, along with the recruitment and infiltration of the vascular wall by circulating leukocytes (Fig. 3).^80^ Potential therapeutic approaches for targeting IL-6/IL-6R signaling are listed in Table 7.

**Table 7:** Potential therapeutic tools for targeting IL-6/IL-6R mediated signaling during COVID-19

Potential therapeutic	Formula	Targets	Mechanism of action	Refs
Sarilumab		IL-6	A human immunoglobulin G1 monoclonal antibody.	^195^
Tocilizumab		IL-6R	IL-6R neutralizing antibody	^196^
LMT-28	C_17_H_29_NO_4_	IL-6	A synthetic IL-6 inhibitor that functions through direct binding to gp130	^197^
Ruxolitinib	C_17_H_18_N_6_	JAK1/2	A potent and selective JAK1/2 inhibitor	^115^
JAK-IN-1	C_20_H_24_N_6_O_2_	JAK1/2/3	A JAK1/2/3 inhibitor	^198^
JAK-IN-3	C_18_H_20_N_4_O_3_	JAK1/3	A potent JAK inhibitor	^199^
STAT3-IN-1	C_28_H_29_NO_6_	STAT3	An excellent, selective and orally active STAT3 inhibitor	^200^
STAT3-IN-3	C_27_H_26_BrN_3_O_6_S	STAT3	A potent and selective inhibitor of STAT3	^200^

### Complement activation contributes to endothelial activation and dysfunction

Complement (C) is an essential part of the innate immunity that serves as a first line of defense against microorganisms.^108^ Complement system comprises of over 30 components, containing membrane-bound regulators, receptors, and numerous plasma proteins. It is well known that complement can be activated through the classical, lectin, or alternative pathways.^109^ The role of complement in the pathogenesis of COVID-19 is to attract more attention.^110^ The complement activation has been confirmed in the pathogenesis of COVID-19, and excessive complement activation leads to acute and chronic inflammation, endothelial dysfunction, and thrombus formation.^111,112^ Activation and amplification of complement generates various potent effectors. Among these effectors, C5a serves as the dominant effector in signaling danger, and the induction of immune modulatory responses.^109^ As the strongest anaphylatoxin, C5a can recruit neutrophils and other leukocytes to the site of activation and prime them through binding to C5a receptor 1 (C5aR1), promoting VE-cadherin degradation. VE-cadherin degradation further results in disruption of the endothelial barrier.^108,109,113^ The membrane attack complex (MAC/C5b-9) of complement has also been reported to play an important role in endothelial dysfunction during immune complex vasculitis.^114^ In addition to C5a and MAC/C5b-9, C3a has been demonstrated to upregulate adhesion molecules (ICAM-1 and VCAM-1) in ECs through p38 MAPK and NF-κB activation (Fig. 3). A recent report indicates that the plasma levels of sC5b-9 and C5a are elevated in COVID-19 patients, and complement activation has been suggested as a novel therapeutic target.^112,115^ Relative agents that can suppress complement activation are listed in Table 8.

**Table 8:** Potential therapeutic tools for modulating complement activation, VEGFA/VEGFR2 Pathway, HSP90 and HIF-1α

Potential therapeutic	Formula	Targets	Mechanism of action	Refs
Anti-C5a monoclonal antibody		C5a	A human monoclonal antibody	^115^
PMX-53	C_47_H_65_N_11_O_7_	C5aR	An active C5aR antagonist	^201^
SB290157 trifluoroacetate	C_24_H_29_F_3_N_4_O_6_	C3a	A selective C3aR antagonist	^202^
PMX 205	C_45_H_62_N_10_O_6_	C5aR	A C5aR antagonist.	^203^
Complement C5-IN-1	C_24_H_32_N_2_O_6_	C5	A small-molecule inhibitor of C5	^204^
SU5408	C_18_H_18_N_2_O_3_	VEGFR2	A potent and cell-permeable inhibitor of VEGFR2 kinase	^205^
GW768505A	C_27_H_19_F_4_N_5_O_3_	VEGFR2	A potent inhibitor of VEGFR2	^206^
Bevacizumab		VEGF	A humanized monoclonal antibody, specifically binds to all VEGFA isoforms with high affinity	^207^
Ramucirumab	C_285_H_434_N_74_O_88_S_2_	VEGFR2	A recombinant human monoclonal antibody that binds to the extracellular binding domain of VEGFR2 and prevents the binding of VEGFR ligands	^208^
Alvespimycin	C_32_H_48_N_4_O_8_	HSP90	A potent inhibitor of Hsp90, binding to HSP90	^209^
Retaspimycin Hydrochloride	C_31_H_46_ClN_3_O_8_	HSP90	A potent and water-soluble inhibitor of HSP90	^210^
TAT-cyclo-CLLFVY TFA	C_116_H_176_N_32_O_33_	HIF-1, VEGF	Inhibit hypoxia-induced HIF-1 activity, and decreases VEGF expression in vitro	
Gramicidin A	C_99_H_140_N_20_O_17_	HIF-1α	Induce degradation of HIF-1α.	

### VEGFA/VEGFR2 signaling-mediated endothelial activation and dysfunction

VEGFs, secreted by a range of cells, are well known for their participation in orchestrating the development and maintenance of blood vascular systems.^116^ They bind to their cognate tyrosine kinase VEGF receptors (VEGFRs) in ECs to elicit various effects.^116^ A large body of evidence suggests that an increase in VEGF induces VEGFR2 activation through ERK1/2 and calcium signaling in ECs.^116^ VEGFA-stimulated VEGFR2 activation is an important process for modulating multiple biological responses, such as proliferation, survival, migration, and permeability.^116^ VEGFA/VEGFR2 recruits the TSAd adapter protein complex, which regulates VEGFA-induced activation of Src tyrosine kinase and vascular permeability in blood vascular ECs (Fig. 3).^116^ Notably, VEGFA is upregulated in the lungs of infections who have died from COVID-19.^63^ Collectively, targeting the VEGFA/VEGFR2 pathway is a possible therapeutic strategy for treatment of COVID-19. Potential therapeutic approaches for targeting VEGFA/VEGFR2 signaling are listed in Table 8.

### CRP promotes endothelial activation and dysfunction

CRP is a major acute-phase protein, and pentamer CRP (pCRP) and monomer CRP (mCRP) are two of its subunits. Its circulating concentration is dramatically elevated at the onset of inflammation and infection. An increase in CRP is correlated with a poor prognosis of COVID-19.^117,118^ Recent studies have suggested that CRP plays a significant role in vascular inflammation and injury, which can damage ECs in vivo and in vitro.^119^ mCRP can promote endothelial cell damage and apoptosis via the p38 pathway.^120^ CRP potently suppresses eNOS transcription in ECs and destabilizes eNOS mRNA, leading to endothelial dysfunction (Fig. 3).^121^ CRP has been demonstrated to upregulate adhesion molecules, facilitate EC apoptosis, and inhibit angiogenesis while augmenting CD14-induced endothelial activation.^122,123^ CRP also potently upregulates NF-κB, a key nuclear factor that can promote the transcription of inflammatory genes.^123^

### Other pathways involved in endothelial activation and dysfunction

Endothelial activation and dysfunction are caused by the combined actions of inflammatory mediators, leukocyte adhesion, and oxidants. In addition to the aforementioned mechanisms, heat shock protein (HSP) 90^60^ and hypoxia-inducible factor-1 (HIF-1)^124^ have also been suggested recently. HSPs belong to a group of highly conserved families of proteins expressed by all organisms, and their expression may be constitutive or inducible. HSPs are commonly considered protective molecules against different types of stress, such as oxidants, toxins, heavy metals, free radicals, and viruses.^125^ As mentioned earlier, a decline in the bioavailability of NO can cause endothelial dysfunction.^23^ The availability of NO to the vasculature is regulated by eNOS activity, and the involvement of HSP90 in the regulation of eNOS activity has been confirmed.^126^ The inhibition of HSP90 can prevent endothelial dysfunction.^127^ Fatal ARDS represented as hypoxia is the leading cause of death among COVID-19 patients.^124^ Local lung hypoxia is predicted to increase the transcription of HIF-1α, and in turn, HIF-1α signaling causes endothelial dysfunction.^128^ HIF-1α has been considered a target for treatment of COVID-19.^124^ Existing pharmacological modulators that act directly or indirectly on HSP90 and HIF-1α are listed in Table 8.

## Conclusions and perspectives

The COVID-19 pandemic has posed an unprecedented challenge to the healthcare community. As our understanding of COVID-19 pathogenesis, endothelial activation and dysfunction are widely proposed by the international medical community. In this review, we summarized possible mechanisms of endothelial activation and dysfunction-mediated inflammation and abnormal coagulation based on clinical findings, suggesting that immunological and physiological functions of ECs, and multiple cellular signaling-mediated endothelial activation and dysfunction should be given more attention. How this will inform specific anti-inflammatory treatments, thus far rather generically targeted, will be another field for proceeding investigation and innovation. Here, we have summarized the critical roles of ECs in the inflammatory process and detailed several mediators and signaling pathways in this cell type that contribute to inflammation. Recently, a lot of agents have been developed to control endothelial inflammation, usually with leukocytes and endothelial activation or dysfunction as the intended targets. The precise therapeutic mechanisms of the medications or monoclonal neutralizing antibodies recommended in this review should be confirmed in future clinical practice, and the efficacy of anticoagulants needs to be verified in well-designed clinical trials. At present, a bulk of clinical and research data cannot be roughly interpreted.

To date, the pathogenesis of COVID-19 mostly remains unclear. The knowledge of the mechanisms of endothelial activation and dysfunction can be used to understand the pathogenesis of COVID-19. Uncontrolled inflammation is the common feature of severe COVID-19. Meanwhile, more attention should be paid to non-traditional forms of inflammation, as therapeutic tools will likely be extremely different for these pathways. For instance, endothelial inflammation has been rarely reported in the pathogenesis of many infectious diseases, but may be much more significant than we know. At last, as we present and interpret this evolving knowledge base, we need to find out which approaches to prevention and treatment of COVID-19, in this context, are most practicable and cost-effective. A collaborative effort between clinicians and biomedical investigators is urgently required to translate the present understanding of endothelium-promoted inflammation to COVID-19 treatment.
